# Euclide, the crow, the wolf and the pedestrian: distance metrics for linguistic typology

**DOI:** 10.12688/openreseurope.16141.1

**Published:** 2023-06-21

**Authors:** Matías Guzmán Naranjo, Gerhard Jäger

**Affiliations:** 1 Linguistics, Albert-Ludwigs-Universitat Freiburg, Freiburg, Baden-Württemberg, 79085, Germany; 2 Seminar für Sprachwissenschaft, Eberhard Karls Universitat Tubingen, Tübingen, Baden-Württemberg, 72074, Germany

**Keywords:** Typology, distance metrics, topographic distance, walking distance, linguistic geography

## Abstract

It is common for people working on linguistic geography, language contact and typology to make use of some type of distance metric between lects. However, most work so far has either used Euclidean distances, or geodesic distance, both of which do not represent the real separation between communities very accurately. This paper presents two datasets: one on walking distances and one on topographic distances between over 8700 lects across all macro-areas. We calculated walking distances using Open Street Maps data, and topographic distances using digital elevation data. We evaluate these distances. We evaluate these distance metrics on three case studies and show that topographic distance tends to outperform the other distance metrics, but geodesic distances can be used as an adequate approximation in some cases.

## 5.1 Introduction

Studying language contact, spatial diffusion, and typology (among others) requires having reliable distances measurements between linguistic communities. However, most work so far has used either Euclidean or geodesic distances (see Guzmán Naranjo & Becker, 2021; Murawaki & Yamauchi, 2018; Ranacher *et al.,* 2021, for an example). Both these approaches, however, make some relatively simplified and unrealistic assumptions about the spatial separation of human populations. Euclidean distances assume that the earth is flat, and geodesic distances assume that the surface of the earth is a smooth sphere. While these assumptions can be warranted in some situations (e.g. communities which are very close together, or in individual islands), using these metrics leads to bias estimates of the separation of speech communities.

As a way of addressing these issues, a method for calculating approximate walking distances was recently proposed by Wichmann & Hammarström (2020), who propose a computationally efficient technique. This method, however, is not exact because it does not attempt to actually follow known walking pathways, but rather, uses population centres to route the paths. Another recent alternative for a computationally efficient approximation is proposed by Kaiping (2021), who calculates exact walking distances between the centre of geographic nodes (hexagons). Each node has an area of roughly 78 square km. Distances between languages are then calculated as the distance between the centres of these hexagons. While impressive, this method is also not exact, and we are not aware of evaluations of how good the resulting distances are for linguistic purposes.

In this paper we do several things. Our main aim is to provide a resource in the form of distance matrices, that can be used for typologists and linguists in general to study contact. Second, we explore the question of how different distance metrics compare to each other. While there are several conceptual problems with using Euclidean and geodesic distances, there has been no attempt at quantifying how much better more realistic distance metrics are.

The structure of this paper is as follows. Section 5.2 gives a brief mathematical and computational description of the four distance metrics we will look at in this paper: Euclidean distances, geodesic distances, topographic distances and walking distances. Section 5.4 describes a case study on modelling potential contact with the different datasets. Finally, Section 5.7 concludes the paper.

## 5.2 Distance metrics

### 5.2.1 Euclidean distance

The simplest type of distance metric we will discuss here are Euclidean distances. This is the distance of a straight line between two points in space. Given the points *a* at the coordiantes (x _1_, y _1_) and *b* at the coordinates (x _2_, y _2_), their distance is given by the formula:


$$\;d_{e}(a,b)=\sqrt{{\left(x_{1}-x_{2}\right)}^{2}+(y_{1}-y_{2})}\;(1)$$

This type of distance has been used explicitly ( Guzmán Naranjo & Becker, 2021), but also implicitly, in the form of adding latitude or longitude to a statistical model ( Verkerk & Di Garbo, 2022). The main reason for its relatively widespread popularity is simple: it is the simplest and fasted distance metric we can calculate for two points, and it can be obtained from latitude and longitude information. In terms of modelling, this distance metric also presents some advantages, namely the fact that popular Bayesian software like STAN or INLA provide ready-made solutions that make very efficient use of Euclidean distances for spatial models. ^1^

Despite these advantages, there are several potential downsides. This distance metric assumes that the points in question lie on a plane, but this is evidently not true for two populations on the earth. For relatively short distances, and distances on the Equator, this is mostly not a problem, especially because we do not expect high accuracy in the centres from which we measure the distances. However, for larger distances, and distance far away from the Equator, Euclidean distance can produce results which are very different from actual distances along the surface of the Earth.

### 5.2.2 Geodesic distance

The geodesic distance, ^2^ or the distance as the crow flies, is the distance between two points on the surface of a sphere. ^3^ Given that the geodesic distance takes into account the curvature of the earth, it is a likely better representation of the separation of two populations. Nonetheless, it also makes several simplified assumptions about the topography of the space between two points. Most importantly, the geodesic distance assumes a smooth surface, without hills, valleys or any other topographic barrier. While this assumption may be justified for cases like the steppe or island archipelagos, it is likely overly optimistic in places with very rugged terrains, mountain ranges, and similar geographic features.

Computationally, this distance is unproblematic, and we will not discuss the technical aspects any further. We calculated the geodesic distance between all lects in Glottolog with the geodist package ( Padgham & Sumner, 2021) in R. ^4^

### 5.2.3 Topographic distance

The topographic distance (or how the wolf runs) is the shortest distance between two points, but we include the elevation changes in between both points. This is the distance along an uneven, rugged surface. To calculate topographic distances we used the gdistance package ( van Etten, 2017) in R. Given its computational challenges, some words on the matter are necessary at this point.

Calculating topographic distances requires building an incidence matrix (a graph of connections between all points) with a digital elevation model (DEM) raster of the region containing the points of interest. Thus, the first step is to assemble a DEM for the region of interest, which, in our case, is the whole world. There are many sources for elevation data freely available, but not all datasets cover the whole planet (the northern most and southern most areas are often missing). For this we used the Global Multi-resolution Terrain Elevation Data 2010 ( Danielson & Gesch, 2011), which does cover the whole globe, and is available at different resolutions (30-, 15-, and 7.5-arc-seconds). While ideally one would use the highest resolution possible, this is not computationally feasible for large areas. We have access to a High Performance Computing server with about 800 GB of ram, but found this was not sufficient to build the incidence matrix for any macro area at the 7.5- or 15-arc-second resolution. For this reason, we used the 30-arc-second resolution of the data, ^5^ which roughly corresponds to about 1 square km per pixel (i.e. we cannot consider elevation changes that cover less that 1 km).

Given a DEM, we can calculate the incidence matrix as the transitions between any two points in the map. The transition between two points is given by √( *h* ^2^+ *v* ^2^) where *h* is the horizontal distance between two points, and *v* the elevation difference. With the incidence matrix for the whole DEM, we can then calculate the shortest path between two points using Dijkstra algorithm, or any other similar algorithm to find the shortest path between two nodes in a graph (see Wang, 2020, for a more in-depth explanation).

However, even at a relatively low resolution, calculating topographic distances is still very resource intensive. Given these computational challenges, we only calculated distances between languages within different macro-areas. Additionally, for North America and Eurasia, we were not able to compute the incidence matrix for the whole macro-area and had to divide these into four, partially overlapping quadrants, and calculate the distance between languages for each quadrant. After having the distances for all points within each quadrant, we propagated the distances across quadrants using points in the overlapping regions.

A recent paper worth mentioning here is Koile *et al.* (2022), which makes use of travel-cost distances. The method used by the authors is similar to the one we present, but it attempts to calculate the travel time using a function to approximate hiking times, instead of taking the actual distance directly. In their study, the authors calculated the travel distances for 77 languages in the Americas, so it does not really represent the type of data we are trying to build in this study.

### 5.2.4 Walking distances

For the purposes of this paper, the walking distance between two points is the distance along mapped roads, walkways and paths that connect those two points. The idea is that road networks are a close representation of the spatial separation between populations because they are the actual pathways used for communication between communities.

For this paper we are using the Open Stree Maps dataset ( OpenStreetMap contributors, 2017) which contains information on roads and other infrastructure for most of the world. ^6^ For the routing we use the OSRM routing engine ( Luxen & Vetter, 2011).

There are, however, some pitfalls calculating walking distances with this approach. These difficulties come from lack of connectivity between points on the map. This lack of connectivity arises in the case of islands which are not joined to the mainland by ferry, ^7^ and some locations without roads or other transitable pathways. The later situation was especially present for languages in the Amazon.

Currently, we do not have any good way to solve this issue. A workaround one can take is to fill in the gaps by using geodesic distances when there are no roads. This approach should provide reasonable results for islands (given that communication between islands and the mainland would have been mostly as direct routes on ship), but it is only a rough approximation for unconnected points in the jungle.

We provide two datasets for the walking distances. One dataset has missing connections for these cases, and the other dataset tries to fill in these missing connections with a simple algorithm which sequentially connects the whole network to the nearest (by geodesic distance) off-network point.

Regarding computational issues, two observations are important. First, OSRM cannot build a graph (the data structure needed for the routing) for the whole world, which meant we had to work on each macro-area individually. Second, in computational terms, we found that the preprocessing steps to prepare the Open Street Maps data took a couple of weeks, but having built the OSRM graphs, calculating the actual distances is extremely efficient. Generally speaking, walking distances are easier to calculate than topographic distances on a moderately powerful server.

### 5.2.5 Taking stock

**Figure 1.**
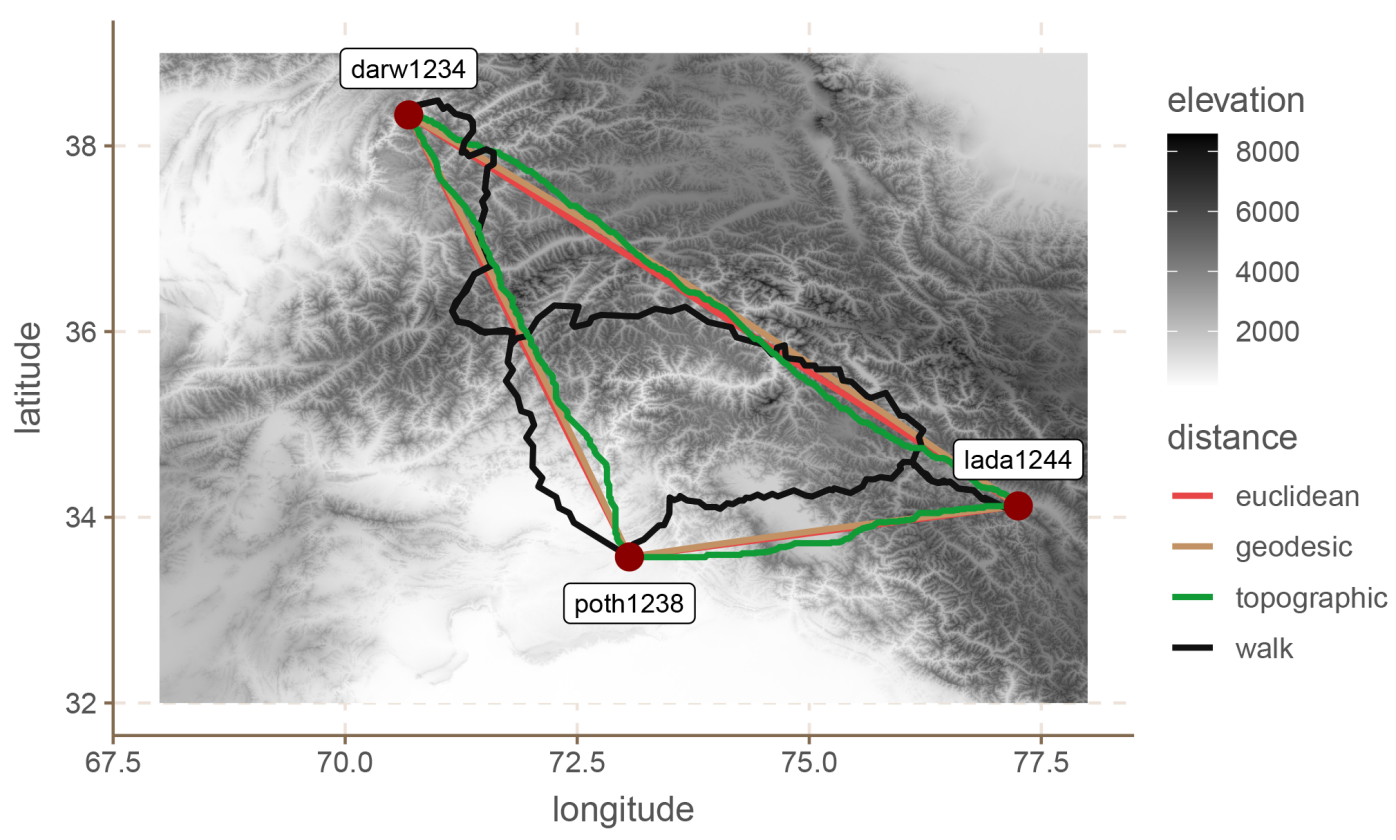
Three Hindu-Kush languages.

So far we have discussed four possible ways of calculating the distance between two points. It is useful to compare what the actual paths look like for the different distance metrics. Figure 1 provides an example of the paths between three points (the locations of three languages) in the Hindu-Kush area (see next section) for the four distance metrics. It is clear that the topographic, Euclidean and geodesic distances are relatively similar to each other, with the topographic path being less straight than the other two. However, the walking path is very different from the other three, and it takes a less straight route.

In computational terms, the topographic distances are the most challenging to compute. They require a considerable amount of resources, and can take several weeks for a single macro-area. Both the Euclidean and geodesic distances are the most efficient, and the walking distances sit somewhere in the middle.

The next three sections present three case studies in which we use these distance metrics to predict grammatical features of languages. The idea of these studies is not to gain linguistic insight, but to evaluate the predictive performance of the different metrics discussed here. To keep the models as simple as possible we will not consider any covariates outside the spatial term.

## 5.3 Evaluation: materials and methods

### 5.3.1 Datasets

We evaluate the four distance approaches in three datasets: Hindu-Kush ( Liljegren *et al.*, 2021), South America ( Carling *et al.*, 2018) and Europe ( Moran & McCloy, 2019). The choice of dataset was partly opportunistic, and partly guided by theoretical considerations. Each case study presents a more detailed overview of each dataset, but in general terms each of these datasets comprises languages annotated for multiple binary features. This is important because in this study we limit ourselves to logistic regression to make all comparisons equal. ^8^. Second, we chose three areas for which there are both interesting topographic features, and at least some note in the literature about contact effects.

In terms of types of linguistic features, the Hindu-Kush data combines phonology, lexical and syntactic features; the South America data exclusively comprises word order features; and the European data is made up of phoneme inventories. It is not our aim with this paper to explore any of these areas or datasets in depth. Our aim is simply to demonstrate how different distance metrics can lead to very different results when modelling contact.

### 5.3.2 The model

There are many different alternatives to model spatial relations from estimating simple correlations ( van Gijn *et al.*, 2017) via autoregressive models ( Murawaki & Yamauchi, 2018) to Gaussian Processes (GP) ( Guzmán Naranjo & Becker, 2021; Guzmán Naranjo & Mertner, 2022). In this paper we use the latter for two reasons. First, GPs can be implemented fairly easily with Stan ( Carpenter *et al.*, 2017), and second, they can use a distance matrix directly. ^9^ PGs are built around a kernel function which transforms the distance matrix into a covariance matrix. There are many alternatives for kernels, but we use a simple exponential kernel (see Duvenaud, 2014, for an in-depth discussion of different kernels).

In this paper we will model each feature independently from the other features. That is, we will predict each feature *f _i_* with a logistic regression with a GP as predictor. ^10^ We predict each feature individually, but there are some possible alternatives to look at all features simultaneously which could be preferable under certain circumstances (see Guzmán Naranjo & Mertner, 2022, for an example using Multiprobit models).

The model definition is as follows:


YBernoulli(inv_logit(μ))(2)

μ=α+η(3)

αNormal(0,1)(4)

$$\;\eta \sim \;MultiNormal(0,\sum_{GP})\;(5)$$

$$\;\sum_{GP}=\;K(x|\lambda ,\delta ,D)\;(6)$$

λInverse_Gamma(3,5)(7)

δNormal(0,3)(8)

$$GP({)}_{j,i}(\lambda ,\delta ,D)=\;\delta^{2}exp\left(-\frac{D_{j,i}^{2}}{2\lambda^{2}}\right)+\delta^{2}\;(9)$$

                                                                                                                                                                                                                               (10)

Where *α* is the model intercept, *λ* is the length-scale of the GP, *δ* the standard deviation of the GP, and *D* is the distance matrix. *K* is the covariance function that transforms the distance matrix into a covariance matrix. The length-scale controls the distance at which two observations can influence each other significantly (a longer length-scale means a longer distance), and the standard deviation of the GP controls how strong the spatial variation can be.

To evaluate the model performance we use 10-fold cross-validation. We split the dataset into 10 groups, train the model using 9 of those groups, and predict the left out group. We then repeat this for all groups. Since we are dealing with binary features, we use balanced accuracy to measure the performance of the classifier. A balanced accuracy of 0.5 means that the classifier is performing at random chance, and thus we can conclude that there is effectively no spatial pattern to the feature in question. A balanced accuracy below 0.5, indicates an issue with the model or distance metric used. A balanced accuracy above 0.5, shows that the there is some spatial structure to the features in question, and that the model can pick up on it and use it to predict the values of the left-out observations.

## 5.4 Case study: The Hindu-Kush

### 5.4.1 Materials

**Figure 2.**
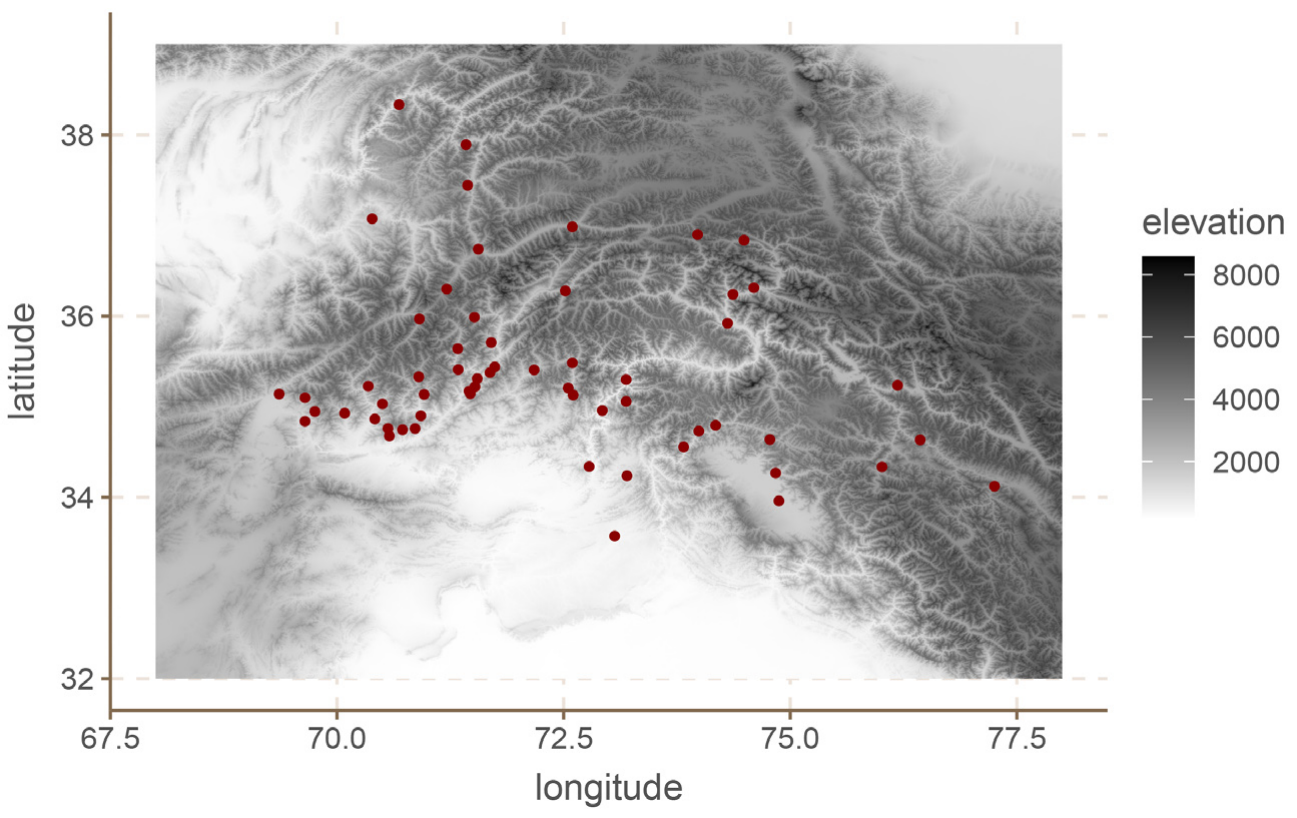
European languages.

This section presents a case study with languages of the Hindu-Kush. We use a dataset by Liljegren *et al.* (2021) which contains 59 languages for the Hindu-Kush area. Figure 2 shows the location of the languages in question.

The original dataset includes annotations for 80 binary features, from phonology to syntax. Since the values of many of these feature were identical for all or almost all languages, we removed features with fewer than 10 or more than 49 positive values. This left us with a total of 48 features. ^11^ Some features have missing values for some languages. The model simply ignores those languages in the case of missing values.

Looking at the Hindu-Kush region is particularly interesting for two reasons. First, contact effects have been extensively documented for this area ( Liljegren, 2019; Liljegren, 2020; Liljegren, 2022, and references therein), ^12^ which leads us to expect positive results at least to a certain extent. Second, and perhaps more importantly, the region is extremely mountainous as can be seen in Figure 2, which means that simple Euclidean and geodesic distances are likely biased estimates of the actual separation between communities (see also Figure 1).

### 5.4.2 Results

**Figure 3.**
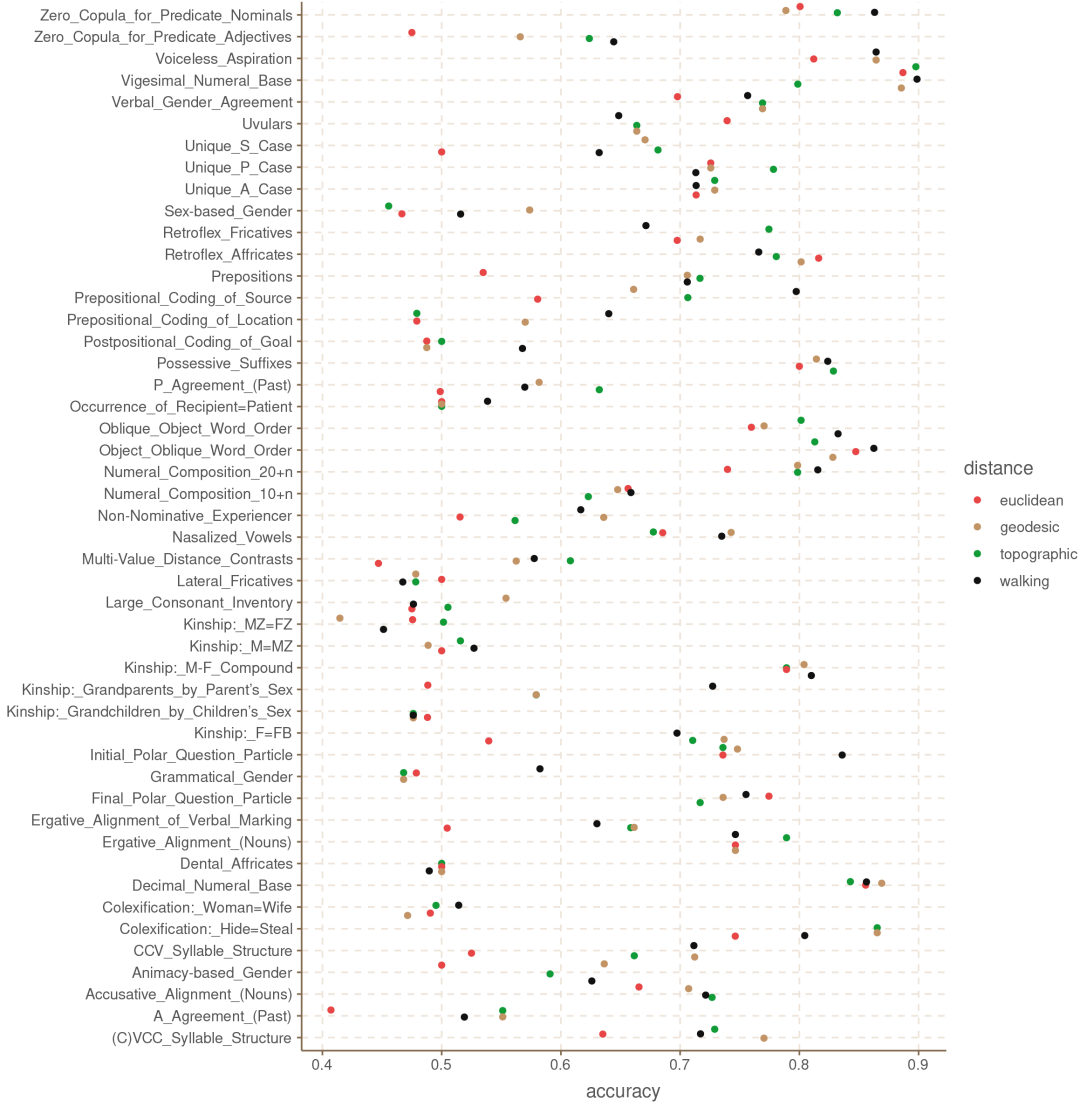
Balanced accuracy by feature and distance metric for the Hindu-Kush.

Figure 3 shows the balanced accuracy of each model for each grammatical feature. This metric is similar to accuracy, but it takes into account the imbalances in the feature values. A balanced accuracy of 0.5 means that the model is performing at random chance, independently of how skewed the distribution of the feature values is.

These results are somewhat unexpected in that they show that there is no clear, systematic winner among the four distance metrics. Even the Euclidean distance, which we could expect to be the least accurate of the four, has the highest accuracy for some of the features like Possessive Suffixes and Oblique Object Word Order. Similarly, the topographic and walking distances perform rather poorly in features like Retroflex Fricatives and P Agreement (respectively). What this shows is that it is not instantly clear that one distance metric is better than the others in all cases.

Table 1 shows the mean accuracy for each distance across all features, and the aggregate counts for how many features each distance produces at the highest accuracy.

**Table 1.** Aggregated accuracy by distance metric for the Hindu-Kush.

distance	mean accuracy	n. times best accuracy	n.times best acc > 0.5
topographic	0.66	16	10
euclidean	0.62	6	3
geodesic	0.67	15	10
walking	0.68	17	9

Going by these results, the walking distance outperforms the other distance metrics in 17 of the 48 features, followed by the topographic distance, then geodesic distance and finally Euclidean distance, which perform considerably worse. In terms of average balanced accuracy, the walking distances also seems to perform slightly better than the others.

We can visualise the differences in the models by plotting the conditional effects of a couple of these models. The conditional effects of a spatial model are predictions of a grid of points on the area in question (from 69 to 77.5 longitude, and from 33.2 to 38.5 latitude, with steps of 0.05, for a total of 18297 points). To build these predictions, we need to calculate the distance from each point in the grid to each of the languages in the dataset. Because walking distances are sensitive to there being an accessible road from the point in question, it is not possible to build the required matrix for the conditional effects of walking distances, at least not for this area of the world. ^13^ For this reason, we only present the conditional effects of Euclidean, geodesic and topographic distances.

For illustration we select the Unique S Case and Zero Copula for Predicate Adjectives, since these two seem to show large differences in the predictive power of the Euclidean distances.

**Figure 4.**
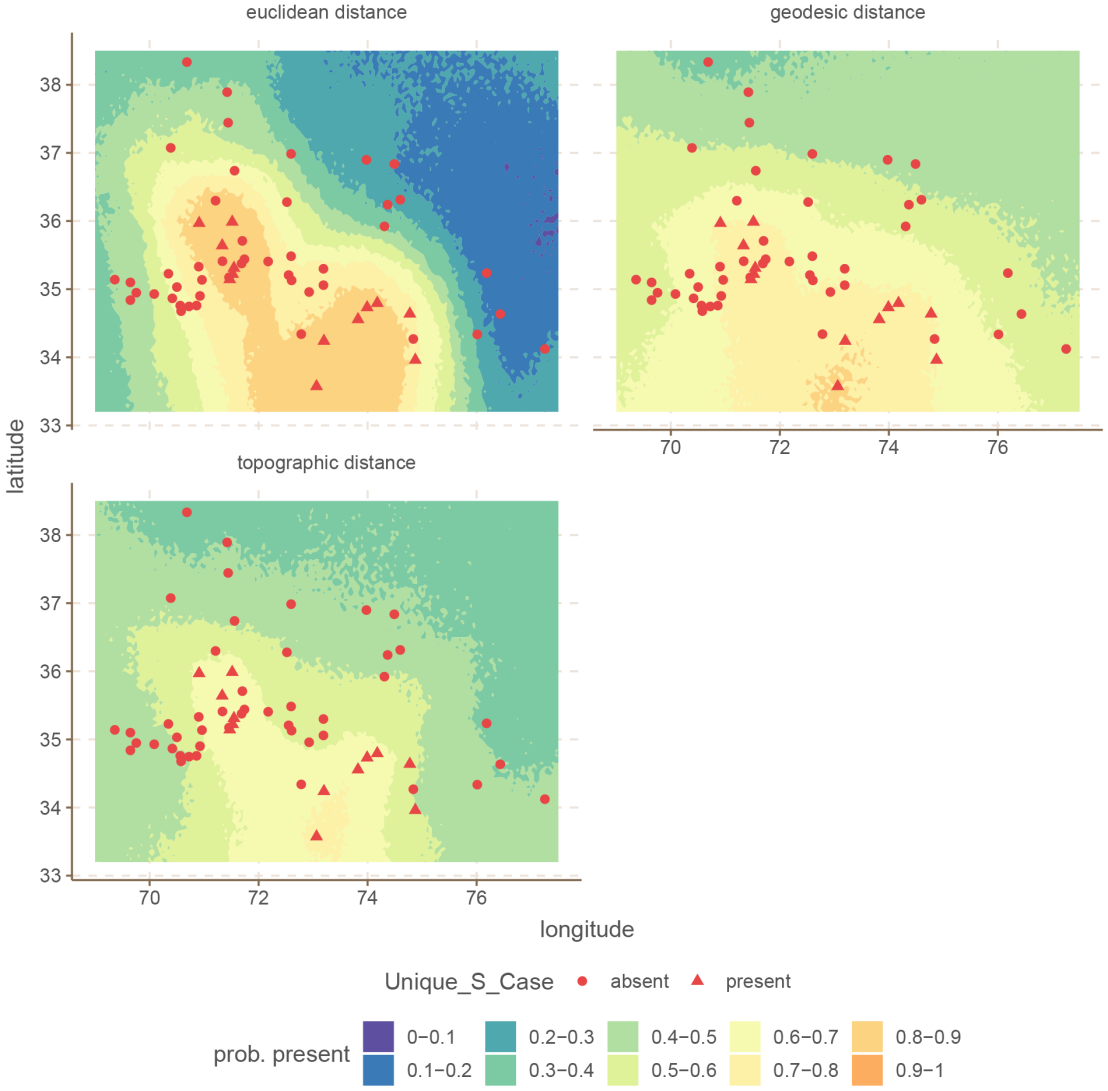
Conditional effects for Unique S Case for the Hindu-Kush.

**Figure 5.**
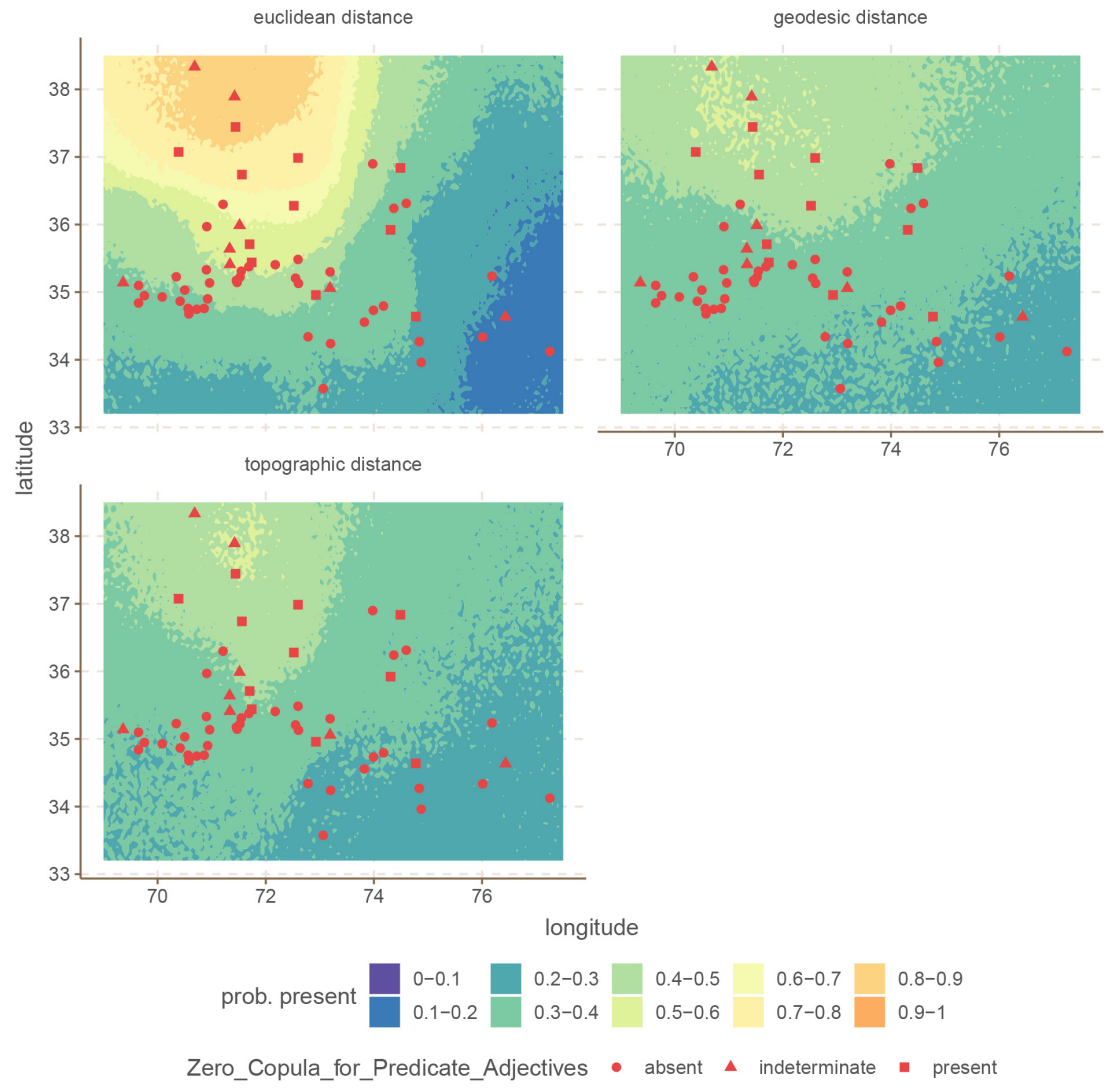
Conditional effects for Zero Copula for Predicate Adjectives for the Hindu-Kush.

These are shown in Figure 4 and Figure 5.

In both cases, the difference is that the Euclidean distance build a much stronger areal effect structure, with more extreme probabilities at the peaks. In contrast, both the areal patterns of the topographic and geodesic distances are smoother, and less extreme. This arises because Euclidean distances are overall shorter than either geodesic or topographic distances, which makes the model infer stronger spatial dependencies. However, in this case, inferring stronger spatial relations leads to overgeneralization and incorrect predictions.

One thing which this modelling approach fails to capture is the fact that a higher accuracy might not reflect the real contact situation. That is, the fact that in some cases the Euclidean distances produced better predictions, does not necessarily mean that the model reflects the actual contact scenario, and it is only finding spurious spatial correlations.

## 5.5 Case study: European phoneme inventories

### 5.5.1 Materials

We now turn to European phoneme inventories. For this case study, we limit ourselves to languages found in the upper left quadrant for Eurasia, between -19.0212 and 82.3004 longitude, and 38.6147 and 68.8326 latitude. This area contains 118 languages in Phoible 2.0. ^14, 15^ Because Phoible lists multiple phoneme inventories for various languages, we randomly chose only one phoneme inventory for each language. We then removed phonemes which were either too rare (present in fewer than 20 languages), or too common (present in more than 88 languages). This left us with a total of 55 phonemes.

**Figure 6.**
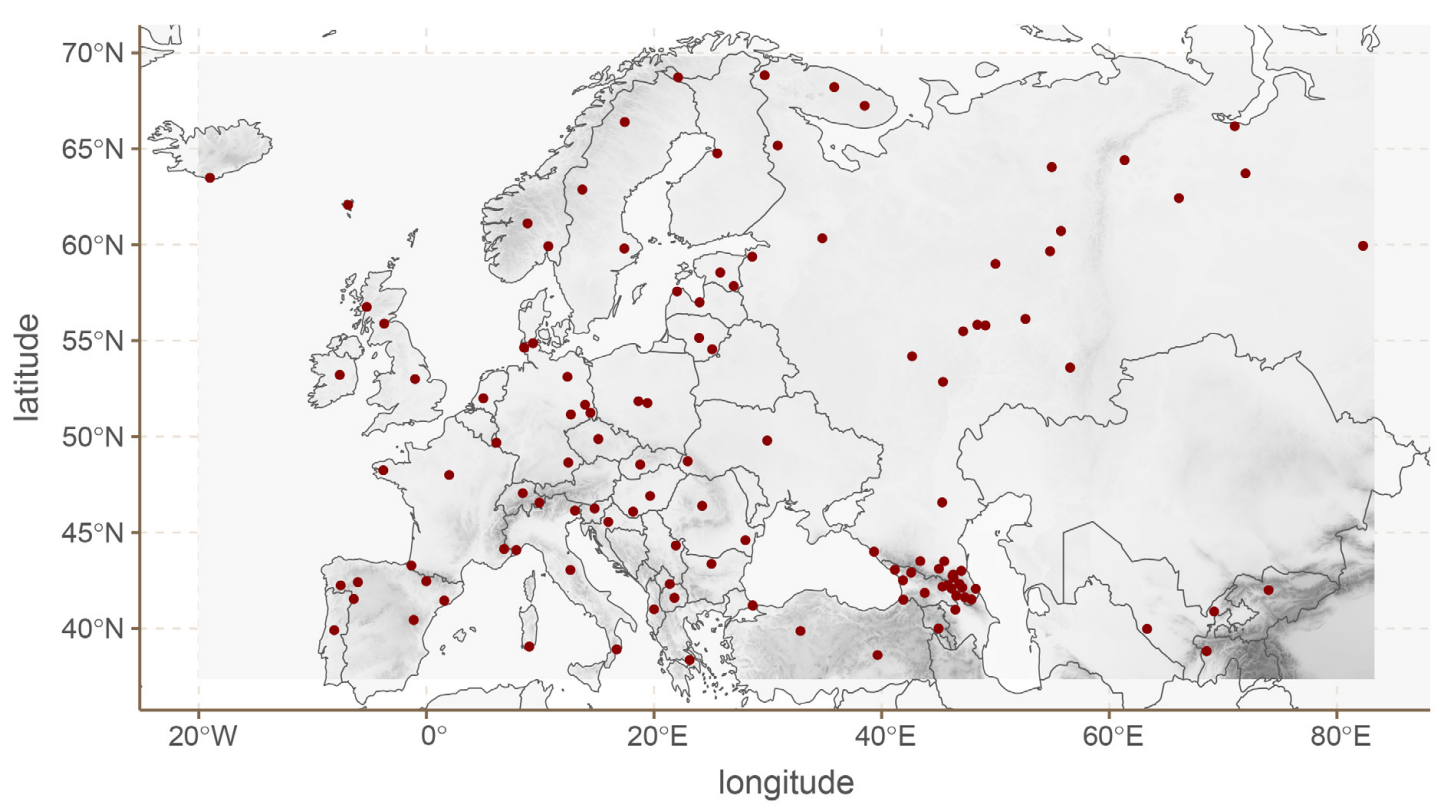
European languages.

Figure 6 shows the distribution of languages in our dataset together with the elevation.

### 5.5.2 Results

**Figure 7.**
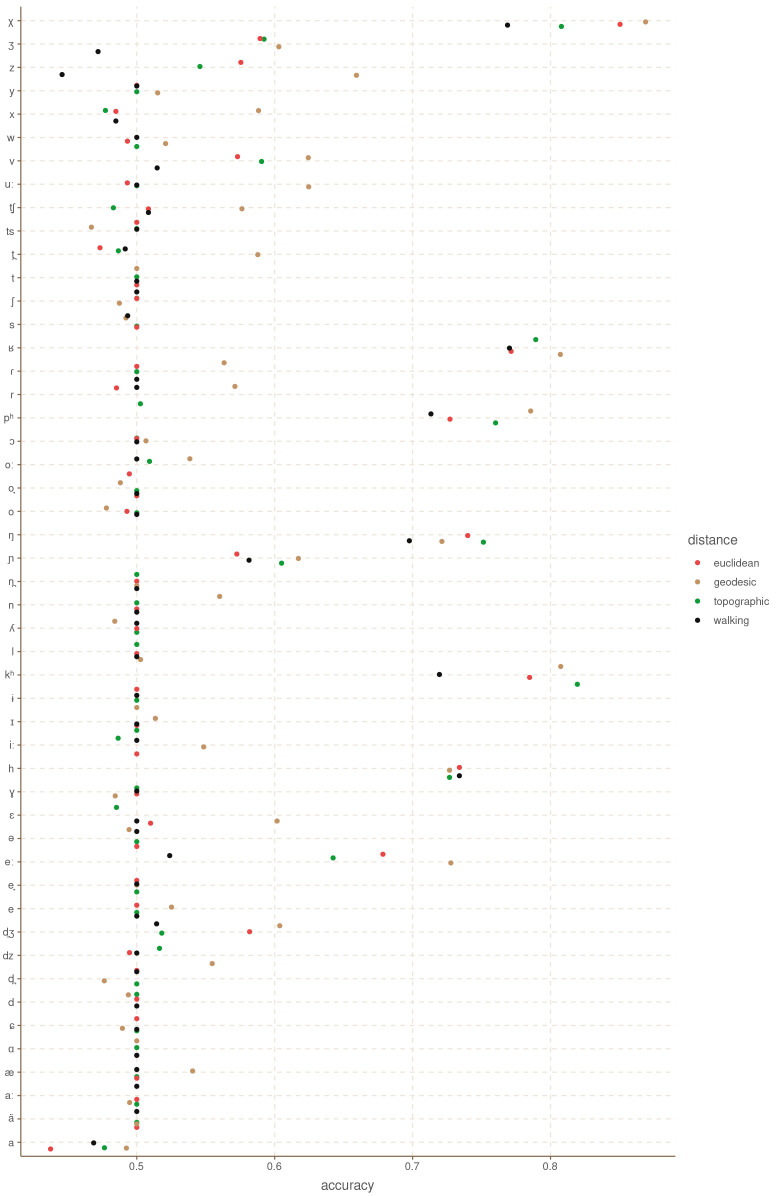
Balanced accuracy by feature and distance metric for Europe.

Figure 7 shows the balanced accuracy for each phoneme. Table 2 shows the mean balanced accuracy, and the number of times each distance metric achieved best accuracy, and best accuracy above 0.5. It is clear in this case that most features are hard to predict, and that they do not show real patterns. However, for those features that do show areal patterns, both the Euclidean and topographic distances outperform the geodesic and walking distance metrics.

**Table 2.** Aggregated accuracy by distance metric for Europe.

distance	mean accuracy	n. times best accuracy	n.times best acc > 0.5
topographic	0.54	20	2
euclidean	0.54	18	1
geodesic	0.57	34	7
walking	0.53	18	1

## 5.6 Case study: South American features

### 5.6.1 Materials

**Figure 8.**
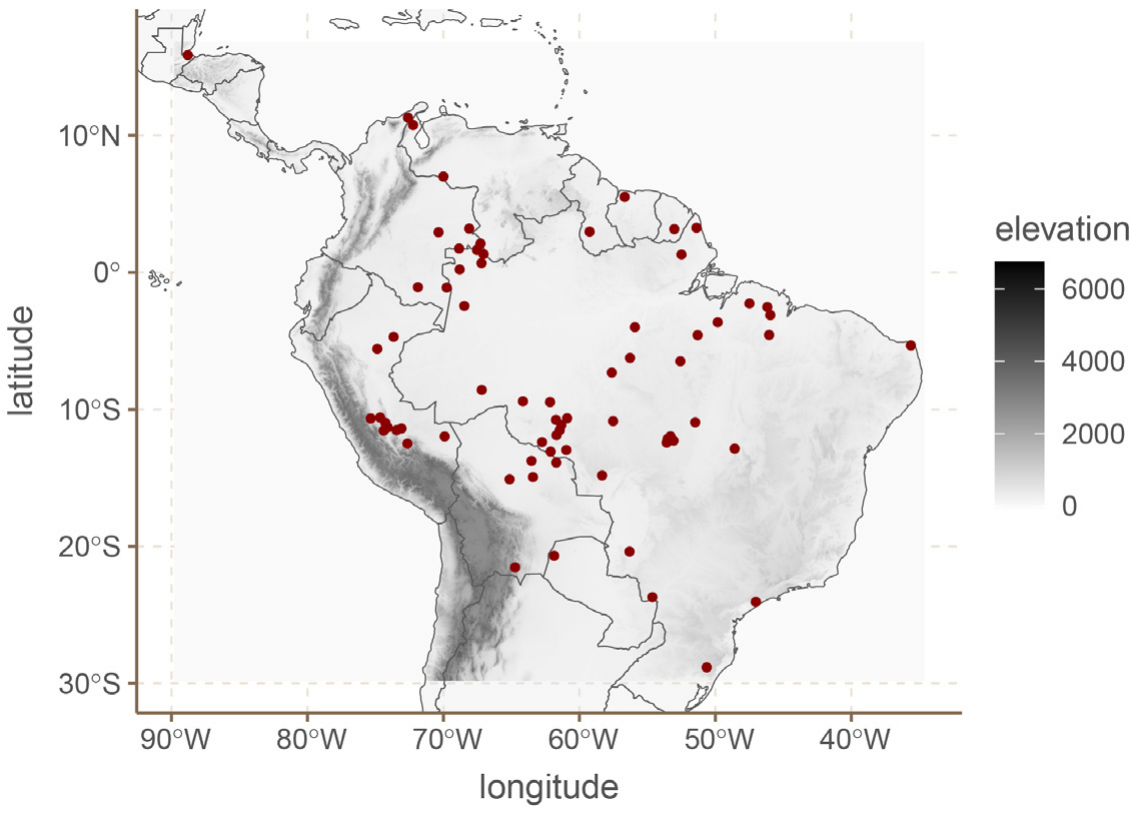
South American languages.

We are using the data for South American languages provided in DIACL ( Carling *et al.*, 2018). This dataset contains data for 70 languages, across 18 binarized word-order features like whether the languages are A(gent)VO or not. As before, we removed features which were either too common (appear in 55 or more languages), or too rare (appear in 15 or fewer languages). The final dataset contains 10 features. ^16^ Figure 8 shows the spatial distribution of the languages in our sample.

### 5.6.2 Results

**Figure 9.**
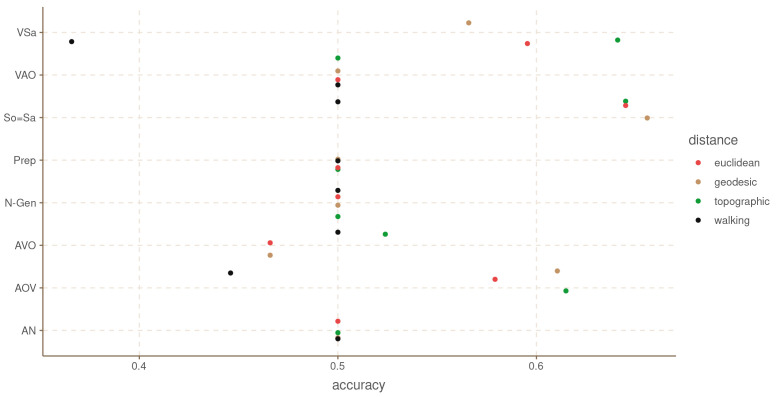
Balanced accuracy by feature and distance metric for South America.

Figure 9 shows the balanced accuracy for each grammatical feature and Table 3 the mean balanced accuracy, and the number of times each distance metric achieved best accuracy, and best accuracy above 0.5. For this dataset we only find evidence of areal patterns for three of the features: VSa, So=Sa and the order AOV. Interestingly, for all three cases, the walking distances had either an at chance performance, or worse than chance. It appears that walking distances for South America are not reliable, or at least not for these data.

**Table 3.** Aggregated accuracy by distance metric for South America.

distance	mean accuracy	n. times best accuracy	n.times best acc > 0.5
topographic	0.55	7	2
euclidean	0.54	4	0
geodesic	0.54	5	1
walking	0.48	4	0

## 5.7 Concluding remarks

We have presented an overview of four distance metrics for typological research, two of which had not been computed before in a large scale. We show that it is in fact possible to compute topographic and walking distances for the worlds languages.

The results in terms of predictive performance are less clear, however. For the Hindu-Kush dataset, the walking distances showed a clear advantage over the other distance metrics, and the topographic distance performed more or less at the same level as the geodesic distance, and the Euclidean distance performed worse of the four. However, for the European dataset, these results are reversed. Both the topographic and Euclidean distances performed considerably better than the walking distance and geodesic distance. Given the fact that the mapping system for Europe in OSM is more developed than for the Hindu-Kush area, we expected the results to be the other way around.

One possible explanation for our results is that walking distances are not very accurate representations of the spatial separation of populations very far apart. Additionally, the Hindu-Kush data has relatively good point accuracy of the location of the languages in questions, while the European data uses very rough approximations. These two factors could be causing the walking distances to perform poorly.

For the South American dataset the results are somewhat more difficult to interpret. It is possible that modern roads and paths are a poor representation of migration paths and trade routes for the languages of the continent. Alternatively, it might just be that our route information for the region is suboptimal.

Overall, we can say for certain that the choice of distance metric can have a very large impact on the models. For some features, we saw upwards of 10% difference between the best and worse distance metric (e.g. Zero Copula for Predicate Nominals in the Hindu-Kush dataset). However, we cannot know a-priori which distance metric will better capture spatial patterns in any one case. From the four distances, the topographic and geodesic distances showed the most consistent performance across datasets, and would be likely to be reasonable first choices. At the same time, in most cases, the Euclidean distances were not much worse than the other distances, and might be a good enough approximation in cases for which performance is critical, or the dataset cover very large areas, and the point-location information is not very precise.

## Data Availability

All distance matrices for both walking and topographic distances are freely available and archived with Zenodo under CC-BY license: 10.5281/zenodo.7973820. The code for building the topographic distances is also available, as well as the code for running the test cases. We also include an environment file which should facilitate replication. See Guzmán Naranjo and Jäger (2023). All Open Street Maps data used for the walking distances calculations can be downloaded from: https://download.geofabrik.de/. We used the versions as of 22.02.2022.

## Footnotes

^1^Particularly in the STAN ecosystem, BRMS provides ready-made functions to compute Gaussian Processes from latitude and longitude information using Euclidean distances, and there are multiple built in covariance functions that use Euclidean distances in the STAN language.

^2^Also referred to as the great-circle distance.

^3^The exact formula for computing the geodesic distance is rather convoluted, and we will not discuss it here.

^4^A middle way between Euclidean and geodesic distances is to use a UTM projection for the data. Using a UTM projection ensures that calculating Euclidean distances on the coordinates produces near correct distances. We will not explore this approach in this paper.

^5^We used the breakline emphasis compression. This approach tries to maintain better sharp changes in terrain elevation ( Gesch, 1999).

^6^We use the data dumps from https://download.geofabrik.de/ downloaded on the 22.02.2022.

^7^While OSM contains ferry information for many places, it is unclear how much information is missing.

^8^This does not mean that either the distances we propose, or the method we describe in the next section only work in logistic regression. In fact, any type of likelihood would work.

^9^With models like those proposed by Murawaki and Yamauchi (2018), one first has to decide on how to build an incidence matrix of neighbours from the distance matrix. This process is not completely straightforward. However, the distance matrices we provide could also be used in those types of models.

^10^There is an important caveat to our modelling. Because of how the routing works, the walking distances do not necessarily satisfy the triangle inequality (in most cases by a couple of meters). The consequence of this is that the estimation of the parameters in the models is biased. Since for this paper we are only looking at predictive performance, this does not matter for our results. However, a researcher interested in understanding the actual spatial structures in the data should take extra care in fixing the distances.

^11^See the supplementary materials.

^12^See also the “Language contact and relatedness in the Hindu-Kush region” project: https://hindukush.ling.su.se/.

^13^A (computationally very costly) alternative would be to mix topographic and walking distances, faulting to topographic distances for cases in which there are no available roads for the walking distances. It is however not obvious how we could go about doing this, and we leave this question open for future research.

^14^ https://zenodo.org/badge/latestdoi/19120525 ( Moran & McCloy, 2019)

^15^An alternative database one could look at is Nikolaev (2018), but we chose Phoibl because it seems to be more popular among typologists.

^16^Unlike the other datasets in this paper, DIACL contains some missing values for some features for some languages. In cases of missing data we simply omitted the languages with missing values for any given feature.
