# Supplementary material for: Euclide, the crow, the wolf and the pedestrian: distance metrics for linguistic typology
Source: Open Res Eur. 2024 Jul 2;3:104. Originally published 2023 Jun 21. [Version 2] doi: 10.12688/openreseurope.16141.2 (PMC11234076; doi:10.12688/openreseurope.16141.2)
Supplement: Peer review history

# Reviewer report: Reviewer response for version 2

**DOI:** 10.21956/openreseurope.18965.r41849

**Published:** 2024-07-09

**Authors:** Francesca Di Garbo

**Affiliations:** 1 Department of Language Sciences; CNRS Laboratory Parole et Langage (UMR 7309), University of Aix-Marseille, Aix-en-Provence, France

I am satisfied with the revisions implemented by the Authors and approve of this version of the article.

Are sufficient details of methods and materials provided to allow replication by others?

Yes

Is the rationale for creating the dataset(s) clearly described?

Yes

Are the datasets clearly presented in a useable and accessible format?

Yes

Are the protocols appropriate and is the work technically sound?

Yes

Reviewer Expertise:

Linguistic diversity, linguistic typology, language contact

I confirm that I have read this submission and believe that I have an appropriate level of expertise to confirm that it is of an acceptable scientific standard.

# Reviewer report: Reviewer response for version 1

**DOI:** 10.21956/openreseurope.17425.r33111

**Published:** 2023-10-05

**Authors:** Ezequiel Koile

**Affiliations:** 1 Department of Linguistic and Cultural Evolution, Max Planck Institute for Evolutionary Anthropology, Leipzig, Germany

This paper explores different methods measuring geographic distances, calculates these distances for all pairs of languages at a global scale, and tests their correlation with linguistic features. It provides a valuable resource for the researchers in linguistic and cultural evolution, and discusses which of these definitions are more suitable for studying different geographic areas and linguistic features.

 I think this is an interesting study which should be indexed after considering the following comments.

**Major comments**

1 - Euclidean distance

 I do not understand why Euclidean distance is considered here, at least with the current definition. I would understand Euclidean distance as a rectangular distance on a plane, where both coordinates (x,y) are defined in the same units. On a spherical surface, the closest equivalent to Euclidean would be geodesic/great-circle distance. Calculating distances like in Eq. (1), just as the squared sum of latitude and longitude in degrees, treating them as distances, is simply wrong. I understand that this calculation is computationally non-intensive, but so is geodesic distance, and much more exact. 

 Euclidean distance as defined here can be useful at relatively short scales, especially near the equator (and it is used in this way inside each area studied in the present paper, except for Northern Europe), but there should be at least some approximation such as the haversine formula, or UTM projection, as mentioned in Footnote 4.

 I disagree with the first statement in the sentence “Euclidean distances assume that the earth is flat, and geodesic distances assume that the surface of the earth is a smooth sphere.” for Euclidean distance as defined in Eq. (1) does not (only?) assume a flat surface of the earth, but a deformed one, where the E-W extension of Greenland at its maximum, from Etah at (long = -72°, lat = +78° ) to Danmarkshavn (long = -18°, lat = +77°) is larger than the maximal E-W extension of South America, from Piura in Peru (long = -80°, lat = -5°) to Natal in Brazil  (long = -35°, lat = -5°), while the first is 1,300km (54°) and the second one 5,000km (45°).

 The authors acknowledge this factː “However, for larger distances, and distance far away from the Equator, Euclidean distance can produce results which are very different from actual distances along the surface of the Earth.”, but still use these metrics.

 The authors mention 4 references, two using either Euclidean or geodesic distances, and then two more using Euclidean distances either explicitly or implicitly. The first two do not use Euclidean distance as far as I understand. Murawaki and Yamauchi (2018) mentionsː

*“Languages were found to be associated with single-point geographical coordinates (longitude and latitude), as shown in Fig. 1. We constructed a spatial neighbor graph by linking all language pairs that were located within a distance of R km.”*

 Which I understand as geodesic distances, since these are measured in km.

 In Ranacher et al (2021):

*“Distance is modelled as a cost function C, which assigns a non-negative value ci,j to each pair of locations i and j. Costs can be expressed by the Euclidean distance, great-circle distance, hiking effort, travel times, or any other meaningful property quantifying the effort to traverse geographical space.”*

 It is not clear to me what they mean by Euclidean distance here, but could not find this used as in Eq.(1) in the further calculation of costs.

 As for the other references, it is true that Guzmán Naranjo and Becker (2021) use it, although pointing out that this is a simplification:

*“For simplicity, we use Euclidean distances between languages. This is, of course, a simplification. It would be fairly easy to use other distance metrics with a GP, e.g. geodesic distances or walking distances (Wichmann and Hammarström 2020).”*

 Finally, in Verkerk and Di Garbo (2022), I do not see this so clearly. Latitude and longitude are used as independent predictors (one would expect that latitude is more determinant for it is more directly related with temperature differences), e.g.ː

*“The languages with the four different types of gender system differ in their mapping onto these four nonlinguistic variables, with the differences between types being larger for longitude and current forest overlap, and smaller for number of L1 speakers and latitude.”*

 In addition, these measures are used in the African continent, which is relatively close to the equator.

 2 - Cross-comparison of case-studies

 I understand that the data available is not completely homogeneous for different areas, but I find it confusing that different linguistic measures are used in the different geographic areasː

*“In terms of types of linguistic features, the Hindu-Kush data combines phonology, lexical and syntactic features; the South America data exclusively comprises word order features; and the European data is made up of phoneme inventories.”*

 I do not see why these different features would be expected to behave in the same way. If there are divergences from the expected correlations with geographic distance, is this because of the topography of the region or because the features studied are affected to a greater/lesser extent by language contact? It would be interesting to test at least one group of features in all 3 geographic scenarios, or split the features in a comparable way (e.g. those in the Hindu-Kush into phonological, lexical and syntactic, to check which ones are more affected by contact).

 More generally, the use of vocabulary like “...topographic distance tends to *outperform* the other distance metrics…” gives the idea of one distance being “better” than others in general. It should be clear in the writing that this outperformance is always a correlation with linguistic features, assuming that shorter geographic distances (in any of its flavors) is responsible for higher similarity among these features among languages.

 3 - Report of main results

 I do not agree with statements such as (Abstract)ː

*“We evaluate these distance metrics on three case studies and show that topographic distance tends to outperform the other distance metrics, but geodesic distances can be used as an adequate approximation in some cases.”*

 Or, more specific outputs for each case study such asː

*“For the Hindu-Kush dataset, the walking distances showed a clear advantage over the other distance metrics…”*

 This is not so clear to me from Table 1. It seems to have performed as well as topographic and geodesic distances.

*“However, for the European dataset, these results are reversed. Both the topographic and Euclidean distances performed considerably better than the walking distance and geodesic distance.”*

 This is also not clear from Table 2. Geodesic distances perform clearly better than the other 3, which perform approximately equally in all 3 metrics.

**Minor comments**

 General:

 Numbering of sections: this is a little confusing along the paper. All sections start with the number 5: 5.1, 5.2,... Unless this is the fifth chapter of a collection, please correct. At the end of the paper, Sections 6-8 appear for Data and Software availability, Author contributions, and Acknowledgments respectively.

 As for the subsections (sections after the 5.), it seems that some of them are incorrectly labeled. For example, at the end of the Introduction, Sections 5.2, 5.4, and 5.7 are mentioned only, while all should be referred: 5.3 is Materials and methods, and I believe 5.4-5.6 should be sub-sections of the same section (Case studies 1, 2, and 3).

 Abstract: 

 Delete the first instance of “We evaluate these distances.” and its corresponding line break.

 Page 3, left column (in the following, P3L):

 Last paragraph Introduction: Rephrase according to new numeration (see above).

 First paragraph Section 5.2:

 (x1,y1) and (x2,y2) : replace 1 → a, 2 → b for consistency with Eq. (1)

 Eq.(1): missing exponent 2 in the second term on the right hand side

 P3R:

 Footnote 3: why is the formula for computing the geodesic distance convoluted? Clarify and add a reference to its definition.

 Last line: “but we include” → “considering”

 P4L:

 First paragraph:

 “northern most” → “northernmost” (same for southernmost)

 “ram” → “RAM”

 Second paragraph: 

 Is this formula correct, symmetric for horizontal and vertical distances? It would be more realistic to add a factor, v → a.v (a >1), and probably test the case studies with different values of a, compared to a = 1?

 Complete the symbol for square root inline (if typographically possible).

 Last paragraph: 

 “... but it attempts to calculate the travel time using a function to approximate hiking times, instead of taking the actual distance directly”. Related to the previous comment, there is only a dimensional factor between time and distance, the latter weighted by a higher cost of moving vertically.

 “77 languages in the Americas” → “languages spoken in 77 villages in the Caucasus”

 P4R:

 1st paragraph:

 “For the purposes of this paper, the walking distance between two points is the distance along mapped roads, walkways and paths that connect those two points. The idea is that road networks are a close representation of the spatial separation between populations because they are the actual pathways used for communication between communities.”

 This might be reasonable for current language contacts, but be aware that in any study involving historic changes, even recent ones, these mapped pathways can differ to a great extent

 2nd paragraph:

 Open Stree Maps → Open Street Maps

 OSRM = Open Street Routing Machine. Define this when first introduced.

 Footnote 4ː Explain the meaning of UTM.

 P5:

 Figure 1:

 It should have a different caption, such as “Example of distances’ comparison for three Hindu-Kush languages”

 Figure 2ː

 The caption should be “Hindu-Kush languages”.

 Figures 1 and 2 (and probably 4 and 5): 

 Add an inset map, it is not very clear what part of the world we are considering beyond the lat and long values.

 Add units for elevation (meters?)

 P5L:

 Last paragraph: “The choice of dataset was…”: dataset → datasets

 P6L:

 First paragraph:

 “...a logistic regression with a GP as predictor.” → as a/the predictor ?

 Footnote 10:

 “...the walking distances do not necessarily satisfy the triangle inequality.”

 Could you please expand on this? If d(A,C) < d(A,B) + d(B,C), why not move from A to C through B instead, reducing the magnitude of  d(A,C) and satisfying the triangle inequality always?

 P6R:

 “from phonology to syntax” → “from phonology and syntax”.

 P7L:

 Top: feature → features

 Figures 3, 7, and 9:

 Add a dashed vertical line at balanced accuracy = 0.5 (all figures have different x-axis scale, so this would help comparability as well).

 Rename the x axis as “balanced accuracy”.

 The coloured dots are not easily readable in the current format. Even in Fig 9, where there are few features in the y-axis, the jittering applied does not help to understand which dot belongs to each feature. I suggest trying a different visualization, e.g. horizontal lines matching all 4 dots for each feature, and different icon shapes for these 4 dots (the colors can be kept besides the shapes). Also, reordering the y-axis by their value in the x-axis (see next item) can help in cases such as Fig 7, with a great proportion of dots on the x=0.5 line.

 Features in each case are ordered in (inverse) alphabetical order. It might be easier to read if they were ordered differently, either by (average? minimum?) value of accuracy, or by some meaningful grouping (e.g. phoneme types in Fig 7).

 Tables 1-3:

 The row order seems arbitrary. Reorder either from simpler to more complex (euclidean, geodesic, topographic, walking) or by some of the values featured, e.g. “n. Times best accuracy”).

 P8L:

 Last paragraph: 

 “from 69 to 77.5 longitude…” add “degrees” or the degrees symbol.

 “Because walking distances are sensitive to there being an accessible road from the point in question,” → “Because walking distances are sensitive to the existence of an accessible road from the point in question,”

 P8R:

 “These are shown in Figure 4 and Figure 5”: I think this sentence belongs to the previous paragraph.

 “...Euclidean distance build…” → “...Euclidean distances build …”

 Figures 4 and 5:

 It is not very straightforward to understand these figures. Why would conditional effects give predictions on presence or absence of a feature? Shouldn’t they only predict similarity among closer locations? If they are based on the distances to each language, isn’t it circular?

 Also, why is the value “Indeterminate” present in Fig 5 but not in Fig 4? If it means NA, it should be included in Fig 4 as well (unless there are no NA’s in this variable). If this is a third category, is this not a binary feature? Also, please be consistent in the choice of symbols (the triangle means present in Fig 4 and indeterminate in Fig 5).

 P8R:

 “...in the upper left quadrant for Eurasia…” Do you mean in the Northwestern quadrant of Eurasia? If so, according to Fig 6, this looks more like Western Eurasia (= Europe mostly).

 Footnote 15: Phoibl → Phoible

 “It is clear in this case that most features are hard to predict, and that they do not show real patterns.” Do you mean areal patterns?

 P9R:

 “...for the worlds languages.” → world’s.

Are sufficient details of methods and materials provided to allow replication by others?

Yes

Is the rationale for creating the dataset(s) clearly described?

Yes

Are the datasets clearly presented in a useable and accessible format?

Yes

Are the protocols appropriate and is the work technically sound?

Yes

Reviewer Expertise:

Linguistic diversity, quantitative methods.

I confirm that I have read this submission and believe that I have an appropriate level of expertise to confirm that it is of an acceptable scientific standard, however I have significant reservations, as outlined above.

# Response

**Published:** 2024-04-05

**Authors:** Matías Guzmán Naranjo

**Affiliations:** Linguistics, Albert-Ludwigs-Universitat Freiburg, Freiburg, Baden-Württemberg, Germany

We have carried out most of the corrections the reviewer suggests. Several formulas were miss-formatted, and several descriptions of the results were not correct, as the reviewer pointed out. We have fixed these. We have also improved several figures according to the reviewer's suggestions. There is one major point the reviewer mentions with which we're not in agreement. Below some specifics: * Regarding the comparison with Euclidean distances: -> There are two things important here. First, and minor, some of the studies like Ranacher et al. do use in practice euclidean distance computations on a particular projection (personal communication with Gereon Kaiping), and in the case of Verkerk and Di Garbo, using the linear latitude and longitude independently, should be equivalent to using euclidean distances as linear predictors on each dimension. There are also some new studies that came out recently that use linear distances like Guzmán Naranjo and Mertner (2022), we have added this reference.  The Second point is technical and comes from two issues we see. First, because brms is gaining popularity as a library for fitting models, it is becoming more and more common for researchers to use the in-built gp() function. This function can only compute euclidean distances. There is no alternative yet. To use other types of distances the researcher needs to write stan code directly, which is still not very widespread. The second factor that plays a role are what’s called approximate Gaussian Processes, which are used for efficiency purposes. While fitting an exact GP to 1000 data points is slow but feasible, it is not possible to fit an exact GP to 2000 data points (at least not in normal research times and consumer hardware). Approx GPs are an alternative that comes very close to estimating the parameters of an exact GP, at the cost of minor exactness. However, approx GPs require covariance functions which are only defined for Euclidean distances (there are also some covariance functions for periodic kernels, but that’s something else). Until someone defines an approx GP for geodesic distances (which is hard), Euclidean distances are the only real alternative here. So, to sum up. We think Euclidean distances are still used, and we do not really see a good reason to not include them in the study. We do not see that comparison as detracting from our main results. We even find that they are an ok approximation in some cases. * Regarding cross-comparisons: -> We do not expect these different features to behave the same way. We are also not trying to compare these features across areas. We only want to illustrate one of several possible uses for the calculated distances, and one of several ways of comparing them with respect to how well they capture the geographic distribution of linguistic features. We have clarified. * Regarding the formula for horizontal and vertical distance: ->  It’s correct. It is possible to use different formulas, but the distances take too long to compute to make this process feasible, at least for us. Additionally, you would actually need to use the angle because steepness is the key factor influencing speed and difficulty of travel. Finding better topographic-style distance is an ongoing project. * Regarding how walking distances calculated on modern pathways may not be valid for past contact: -> Indeed. As mentioned in the paper, we believe this might be one of the reasons that walking distances work so poorly for Europe. We have added a note at this point. * Regarding triangle inequality and OSRM: -> We have expanded a bit. The problem is very technical. The issue comes from how OSRM works internally. The routing algorithm in OSRM follow road constraints like allowed direction of travel as well as vehicular type allowed on the roads. This leads to a combination of factors which result in non-triangular inequality situations. For example, very often going from A to B requires following a single direction road on which there is no sidewalk, and thus, the direction of the road must be respected, which means that A->B and B->A cannot be done on that same route. This sort of situation can be corrected on small matrices with well understood algorithms, but these did not work on our larger matrices. What seems to be happening is that there are too many interacting edges, and if we fix A-B-C, some new issue arises for A-B-D and A-B-E, etc.   The algorithm (and script) we tried:   /*  * Triangle fixing algorithm - implementation of algorithm 3.1  * (Metric_Nearness_L2) in Brickell, J., Dhillon, I., Sra, S., and  * Tropp, J. (2008). The Metric Nearness Problem. SIAM. J. Matrix  * Anal. & Appl. 30, 375-396.  *  * (c) Toni Giorgino  2016  * Distributed under GPL-2 with NO WARRANTY.  *  * $Id: triangleFixing.c 424 2016-08-25 19:45:42Z tonig $  *    */ * Regarding or ME plots: -> The model uses the GP to predict the expected value of a feature at some point, given the distance of that point to the points the model was trained on. It is the same thing with the CV, the difference is that for the CV we predict locations of languages we left out of the training, and for the ME we predict a dense grid of points.

# Reviewer report: Reviewer response for version 1

**DOI:** 10.21956/openreseurope.17425.r33110

**Published:** 2023-10-02

**Authors:** Francesca Di Garbo

**Affiliations:** 1 Department of Language Sciences; CNRS Laboratory Parole et Langage (UMR 7309), University of Aix-Marseille, Aix-en-Provence, France

**Summary and general comments**

 This paper introduces two novel techniques for computing distances between languages. The two distance metrics created through these techniques are a measure of topographic distance and one of walking distance between language communities. The paper illustrates the technical implementation of the two measures and shows how they can be used to study linguistic areality and contact effects from a typological perspective. The accuracy of the two metrics is compared against two distance metrics previously used in typological studies of language contact: Euclidean distance and geodesic distance. The four metrics are applied to three existing typological databases, each focusing on linguistic features of three established linguistic areas: the Hindu-Kush, Europe, and South America. The studies show that the choice of distance metric can significantly affect the type of generalizations that be drawn from a model, and that it is hard to establish a priori which measure would work best to capture areality effects in a given dataset. The results presented in the paper suggest that, in general, topographic and geodesic distance perform most consistently across the three datasets.

 The paper makes an important methodological contribution to the study of spatial effects on the distribution of linguistic diversity. Firstly, because it enriches the range of methods typologists can use to control for areal biases in the distribution of structural properties of languages. Secondly, because the method offers a promising new way to account for areality bottom-up. This is especially crucial not only for the purpose of bias control, but also for detecting linguistic areas and/or areality effects in an exploratory fashion, for instance by testing hypotheses about the areal spread of given linguistic phenomena independently of pre-established areal groupings.

 I would encourage the authors to develop a bit more on this second point, which does not emerge so neatly from the current version of the text, but, which, I believe, is of great potential for linguistic typology. Have the authors thought, for instance, about applying their new measures to the newly published Grambank dataset? This newly released database, the largest worldwide typological database to date, could offer an important test ground to further test the validity of the two distance metrics and, more generally, to detect areality effects bottom-up.

 In addition to the above-mentioned suggestion, I have a number of more minor comments and suggestions of revision, which are highlighted below.

**Minor comments and suggestions**

**Abstract**

*"We evaluate these distance metrics on three case studies and show* *that topographic distance tends to outperform the other distance* *metrics, but geodesic distances can be used as an adequate a* *pproximation in some cases."*

 This passage can be a bit misleading (when compared to the rest of the paper). It seems to imply that topographic distance is consistently better than the other three measures, while, in fact, depending on the model and the phenomena at stake, things can vary. I would suggest the authors to rephrase this passage by framing things in line with what stated in the concluding section (cf. *From the four distances, the* *topographic and geodesic distances showed the most consistent* *performance across datasets, and would be likely to* *be reasonable first choices. At the same time, in most cases,* *the Euclidean distances were not much worse than the other distances, and might be a good enough approximation in cases* *for which performance is critical, or the dataset cover very* *large areas, and the point-location information is not very* *precise.*)

**Page 3**

*"Section 5.4* *describes a case study on*

*modelling potential contact with the different datasets."*

 In the abstract, and in the rest of the paper, the modelling study is presented as a series of three independent case studies. Please rephrase in order to make things consistent.

**Page 6**

 Figure 2. European languages > Hindu-Kush languages

**Page 8, about the interpretation of Table 2 and of the European case study**

*"However, for those features that do show areal* *patterns, both the Euclidean and topographic distances outperform* *the geodesic and walking distance metrics."*

 Is this really the case? Unless I interpret things wrongly, judging from Table 2, it seems that it is the topographic and geodesic distances that outperform the other distance measures in this case. Could you please clarify?

Are sufficient details of methods and materials provided to allow replication by others?

Yes

Is the rationale for creating the dataset(s) clearly described?

Yes

Are the datasets clearly presented in a useable and accessible format?

Yes

Are the protocols appropriate and is the work technically sound?

Yes

Reviewer Expertise:

Linguistic diversity, linguistic typology, language contact

I confirm that I have read this submission and believe that I have an appropriate level of expertise to confirm that it is of an acceptable scientific standard, however I have significant reservations, as outlined above.

# Response

**Published:** 2024-04-05

**Authors:** Matías Guzmán Naranjo

**Affiliations:** Linguistics, Albert-Ludwigs-Universitat Freiburg, Freiburg, Baden-Württemberg, Germany

We have implemented most of the reviewer's suggestions, and rephrased accordingly. Regarding the specific point on exploring areality with Grambank: -> We have and this is currently work in progress. Since it is a bit off-topic we don’t go much into detail. But we now make a brief mention of it.

# Reviewer report: Reviewer response for version 1

**DOI:** 10.21956/openreseurope.17425.r33113

**Published:** 2023-06-29

**Authors:** Dan Dediu

**Affiliations:** 1 Department of Catalan Philology and General Linguistics, University of Barcelona, Barcelona, Catalonia, Spain; 2 Catalan Institute for Research and Advanced Studies (ICREA), Barcelona, Spain

This paper introduces two new ways of computing between-languages distances and provides directly usable datasets for these (plus two “older” methods). Given the general importance of ways of quantifying space in many cross-linguistic (and, more generally, cross-cultural) studies, I think this is an important addition to the literature, especially given the massive computational resources needed to compute one of these new distances, resources that are now readily available to the “average” language scientist.

 However, I do have a few suggestions and questions that might help improve the paper. In their (approximate) order of appearance in the paper:

 Abstract:

- Remove 1 ^st^ “We evaluate these distances.” (and, in general, the papers seems to in need of a careful reading).
- After having read the paper, I think the last sentence is a bit misleading, in that it is far from clear to me (and to the authors themselves, in fact) that “topographic distance tends to outperform the other[s]” in a meaningful and general way – I think this should be toned down as the paper is not about results but about the method and the distance matrices IMHO.

English language:

- Please check prepositions, spelling, plural, etc in: "warranted in some situations"; "leads to bias estimates"; "used for typologists and linguists"; "simplest and fasted distance metric"; "Open Stree Maps"; "show real patterns"; "but we chose Phoibl"; "before in a large scale"; "for the worlds languages"; "The simplest type of distance metric we will discuss here are Euclidean distances" -> “is”?; "the northern most and southern most areas" -> write connected (“northernmost”)?; "distances, faulting to topographic" -> defaulting?; "Euclidean distance build" -> “distances build” or “distance builds”

P3:

- missing ^2 after (y1-y2) in d_e formula.

P4:

- Para “Given a DEM…” -> please reformulate to make understanding this process easier the first time one reads this. My suggestion would be to 1 ^st^ clarify that you compute the graph between all points in the DEM and then you use these to compute the minimum distance between the points one is actually interested in.
- Same para: please justify why sqrt{h^2 + v^2} as it is not immediately obvious why the h and v dimensions should be treated (a) separately and (b) have equal weight. Naively I would have expected something more like sqrt(x^2 + y^2 + w*v^2} where w is an (empirical) weight qualifying the relative “costs” of vertical vs horizontal displacements?
- Para “However, even at a …“ -> I was wondering if this would not work as a general solution in that maybe one can consider overlapping neighborhoods of the points of interest, but of course defining these neighborhoods brings in assumptions about the relative costs of vertical vs horizontal displacements (see above), but I guess it would be something worth discussing in the paper (if not testing)?

P5:

- “PGs are built” -> GPs?

Figure 1 (and figures in general):

- The color palette is atrocious for people with abnormal color perception! It is impossible the distinguish the first 3 distances on the map. Please try to use something like viridis or equivalent.

Footnote 10:

- Please explain why the triangle inequality is important for the GP and how bad their violation is (to footnote hints but I think this needs a better development).

Eq 9:

- The “()” after “GP” are meaningful?

Figure 2:

- That dark red (I guess) for the dots is very hard to see (for me) against the grayscale of the elevation.
- “European” -> “Hindu-Kush”.

P7 (and elsewhere):

- please justify these cut-off points (here, <10 and >49 – especially 49 seems a bit weird).
- 2 ^nd^ sentence and following of 1 ^st^ para of section 5.4.2 “This metric…” -> this is partially repeating stuff that was already mentioned -> please move above.

Figure 3:

- could you also use symbols as well as colors? Highlight the 0.5 vertical line. Order the features in a meaningful way? Connect the same-color points by lines as well (resulting in an easier-to-follow “profile” but make it clear this does not imply any “linkage” between features). For the vertical jitter, either use the same jitter for all features and/or draw alternating colored bands by feature to make it clear which point belongs to each feature (now it is pretty hard to see, especially combined with the color scheme).

P8

- “What this shows is that it is not instantly clear that one distance metric is better than the others in all cases.” -> I think the message is less optimistic here, namely that it really seems to depend on the feature considered, which is interesting in itself.
- Table 1 (and the others): the mean accuracy is hard to judge without some measure of variation (IQR, stddev…) – maybe better show the actual distribution (histogram/density)? (also, they look very similar to my eye – some statistical tsting would be useful?) Also n.times best accuracy is not very informative without knowing by what margin these were “best”.
- Para “One thing which…” I think this is *very* important and should be discussed in the Discussion, together with the idea that languages are not points.
- “languages, we randomly chose only one phoneme inventory for each language.” -> I know this is sometimes done, but it does not mean it is a good idea: sometimes, depending on the question, different inventories for the same language in PHOIBLE give very different answers…
- Again, justify the cut-off points, but here this might be even more important because, as argued for SegBo (see, e.g. Eisen, E. (2019) ^1^) when looking at segment borrowing it is the segments with around 50% frequency that might carry the best signal.

Footnote 16:

- I am confused as I think you mention that for H-K languages there was also some missing data?

P9:

- “For the Hindu-Kush dataset, the walking distances showed a clear advantage over the other distance metrics” -> I am not sure this is the message I got from the results…

Figure 6:

- add elevation legend (also make it clear that the legend’s values are *global*).

Finally, two general comments:

1. Language are not points: I clearly agree with the authors’ take in the paper (I do the same myself) but I think this point should at least be discussed and maybe ways of addressing it wrt the two new distances mentioned (if any)?
2. The right benchmark for these distances: I agree with the authors that this is not the point of this paper, but still choosing the right benchmark might make the difference between these two new distances being actively embraced by the community or lingering in the literature… Given that *most* actual uses of distances is to *control for* (i.e., remove) contact, maybe this would be a more appropriate benchmark? Also, (balanced) accuracy might not be the best (or, at least, the only) quantification of success here, but instead some sort of formal model comparison as well? And, finally, looking at known linguistic areas (e.g., the Balkans, which has non-trivial topography) might help? These are just ideas (and half-digested at that), of course, but I think the authors might want to at least discuss them given the amount of work (and computational power) already expended on this project?

Are sufficient details of methods and materials provided to allow replication by others?

Yes

Is the rationale for creating the dataset(s) clearly described?

Yes

Are the datasets clearly presented in a useable and accessible format?

Yes

Are the protocols appropriate and is the work technically sound?

Yes

Reviewer Expertise:

Linguistic diversity, cross-linguistic studies, statistics

I confirm that I have read this submission and believe that I have an appropriate level of expertise to confirm that it is of an acceptable scientific standard, however I have significant reservations, as outlined above.

## References

[1] : The Typology of Phonological Segment Borrowing. *Thesis for: MA in linguistics. Hebrew University of Jerusalem* .2019; 10.13140/RG.2.2.36245.55529 10.13140/RG.2.2.36245.55529

# Response

**Published:** 2024-04-05

**Authors:** Matías Guzmán Naranjo

**Affiliations:** Linguistics, Albert-Ludwigs-Universitat Freiburg, Freiburg, Baden-Württemberg, Germany

We have corrected most issues the reviewer highlights, and added further explanation where things were not clear, as well as reformulated claims which were too strong/not quite in line with the results. We have also redone the plots to make them more color-blind friendly. There are a couple of things where we do not quite agree on with the reviewer's suggestions, or consider it to be for future work. Here the concrete points: * Regarding ways of calculating travel times/distances: -> There are, of course, many alternative methods to calculate travel costs over a surface with elevation differentials. The formula we use is just Pythagoras theorem, it assumes that to go between two adjacent points, one has to travel in a straight line between them (the hypotenuse). It effectively reduces a complex surface to a surface made up of triangles. The result is a distance that approximates the real total distance traveled. The quality of the approximation depends on the resolution of the DEM. There are alternatives using formulas that approximate walking times which look similar to what you propose. We chose this solution because it avoids having to justify some value for w, which will inevitably change depending on a multitude of factors like terrain unevenness, steepness, flora, etc.. Testing travel time/travel cost metrics is an interesting idea we do plan to pursue in the future. * Regarding the suggestion of overlapping neighbors: -> Our guess is that the reviewer means something like what Kaiping did for the Americas? It’s an alternative method, but then you lose resolution for the distance between the neighbors. Depending on the purpose of the distance, that would make the calculation faster, but we already did most of the heavy lifting here. We are not sure that such an approach would help with higher resolution DEMs. Increasing DEM resolution even by a few arcseconds makes the data unmanageably large. * Regarding statistical testing of the tables: -> We’ve added sd to the tables but we’re not sure adding statistical testing would make much sense. We could add some credible intervals to the plot, but that would only clutter and, we believe, send the wrong message on what we’re trying to do with the paper. Proper, exhaustive comparisons of how these distances would require a whole different paper with a lot more tests. * Regarding randomly chosen phoneme inventories: -> The reviewer is correct. If one is interested in exploring some concrete linguistic question, then randomly choosing inventories might not be the best approach. However, we are only using these datasets as illustration of what can be done with our distances, and do not really think it is worth it to try to find the best dataset choices within phoible in this paper. * Regarding cutoff points: -> That’s precisely what we chose, phonemes which are neither too frequently present nor too frequently absent. * Regarding polygons and points: -> We agree. Languages are not points. Dealing with polygons is part of ongoing research by the first author, and it is still unclear how distances should be measured in such cases (from the centroid? on average? what about overlaps? etc.). But, however one wants to resolve that question, the technical question of how to then measure distances will be related to the methods we suggest here. * REgarding benchmarks and "controlling for": -> Well, technically, “controlling for” and “estimating” are the same thing when building a model. So when comparing model performance, if a distance produces better accuracy in a model like ours, it means it will ‘control for’ contact better, in the sense that it will capture a larger amount of variance due to spatial correlations.  -> In other words. Imagine you’re interested in feature F for some group of languages. You have two options, geodesic distances and topographic distances. If you test the distance metric on its own, topographic distances achieve an accuracy of 0.8 while geodesic distances an accuracy of 0.6. This means that topographic distances capture more information about the spatial distribution and spatial relations in your data. -> What we present *is* formal model comparison, we are just using acc as a metric. We could also use something like ELPD instead, which is often preferred for these types of models. The downside of ELPD is that it’s hard to interpret and not sound given the issues with walking distances and model sampling. This is the mean reason we think accuracy is a better choice in our case.
